# A novel statin-mediated “prenylation block-and-release” assay provides insight into the membrane targeting mechanisms of small GTPases

**DOI:** 10.1016/j.bbrc.2010.05.045

**Published:** 2010-06-18

**Authors:** Bassam R. Ali, Ian Nouvel, Ka Fai Leung, Alistair N. Hume, Miguel C. Seabra

**Affiliations:** a Department of Pathology, Faculty of Medicine and Health Sciences, United Arab Emirates University, Al-Ain, United Arab Emirates; b Molecular Medicine, National Heart and Lung Institute, Imperial College London, London SW7 2AZ, UK

**Keywords:** GTPases, Prenylation, Statin, Trafficking, Rab proteins

## Abstract

Ras super-family small GTPases regulate diverse cellular processes such as vesicular transport and signal transduction. Critical to these activities is the ability of these proteins to target to specific intracellular membranes. To allow association with membranes Ras-related GTPases are post-translationally modified by covalent attachment of prenyl groups to conserved cysteine residues at or near their C-terminus. Here we used the HMG-CoA (3-hydroxy-3-methylglutaryl-coenzyme A) reductase (HMGCR) inhibitor mevastatin to develop a ‘prenylation block-and-release’ assay that allows membrane targeting of prenylated proteins to be visualized in living cells. Using this assay we investigated the cytosol to membrane targeting of several small GTPases to compartments of the secretory and endocytic pathways. We found that all Rabs tested were targeted directly to the membrane on which they reside at steady-state and not via an intermediate location as reported for Ras and Rho proteins. However, we observed that the kinetics of cytosol to membrane targeting differed for each Rab tested. Comparison of the mevastatin sensitivity and kinetics of membrane targeting of Rab23, Rab23 prenylation motif mutants and H-Ras revealed that these parameters are strongly dependent upon the prenyl transferase with Rab geranylgeranyl transferase substrates exhibiting higher sensitivity and requiring greater time to recover from mevastatin inhibition than farnesyl transferase substrates. We propose that this assay is a useful tool to investigate the kinetics, biological functions and the mechanisms of membrane targeting of prenylated proteins.

## 1 Introduction

The post-translational modification of peripheral membrane proteins by lipids is a key mechanism in the regulation of protein targeting and function in all eukaryotic cells. Prenylation is a lipidic post-translational modification involving the irreversible covalent attachment of either farnesyl (15-carbon) or geranylgeranyl (20-carbon) isoprenoids to conserved cysteine residues at or near the C-terminus of numerous cellular proteins [1–3]. Prenylated proteins in eukaryotes include nuclear lamins, Ras and Ras-related small GTP-binding proteins, γ subunit of trimeric G proteins, protein kinases, fungal mating factors and others [4–6]. Prenylation is frequently associated to reversible post-translational modifications or ligand binding events such as palmitoylation, phosphorylation or GTP-binding [5]. All members of the Ras super-family of small GTPases with the exception of Ran, Rit and Sar1 are prenylated [4,6,7]. For Ras and Rho family GTPases, most of their members are singly prenylated by either farnesyl transferase (FT) or geranylgeranyl transferase (GGT), respectively. The determinants allowing recognition of substrate by FT and GGT have been studied extensively [4]. The newly lipidated Ras and Rho proteins are then targeted to the cytoplasmic face of the endoplasmic reticulum (ER) where they undergo further modification including removal of the AAX tripeptide and methylation of the α-carboxyl group of the newly exposed C-terminus prenyl-cysteine [6]. The processed proteins are then trafficked via classical or non-classical secretory pathways to the plasma membrane and may be further modified e.g. palmitoylated and phosphorylated. In contrast most members of the Rab family have two C-terminus cysteine residues and once in complex with chaperone Rab escort protein (REP-1/-2) may doubly geranylgeranylated by Rab geranylgeranyl transferase (RGGT) [4,8,9]. However, there are exceptions among the Rabs that contain only one C-terminus cysteine e.g. Rab13 and 23 and are singly prenylated by RGGT. Also within this subset some singly prenylated Rabs appear to have a bona fide CAAX box e.g. Rab8 C-terminus tetra-peptide CVLL, and may therefore be substrates for either RGGT or GGT [4,8]. Nevertheless *in vivo* it is thought that all Rabs are preferentially prenylated by RGGT [10–14]. This idea is based on the observation that all Rabs contain five conserved sequence elements known as Rab family regions (RabF) that allow their recognition by REP and consequently prenylation by RGGT [15]. After prenylation Rab remains in complex with REP that plays a critical role in targeting Rab to a specific membrane location by mechanisms that are currently unclear [16].

The cholesterol lowering compounds known as statins were discovered through isolation of microbial agents capable of disrupting HMG-CoA (3-hydroxy-3-methylglutaryl-coenzyme A) reductase (HMGCR) activity. HMGCR catalyzes the committed step in cholesterol biosynthesis [17], and is critical to production of ergosterol and isoprenoids e.g. farnesyl (C15) and geranylgeranyl (C20) [18]. Mevastatin was the first of many ‘statin’ compounds found to inhibit HMGCR activity, and these compounds have since gained much attention as cholesterol lowering drugs [19]. Statins also deplete the cell of isoprenoids, essential for functionality of proto-oncogenes such as Ras and Rho, and as a result they have been the subject of research into their potential application as anti-cancer therapeutics [20,21]. Statins competitively inhibit the four-step deacylation of HMG-CoA to mevalonate and CoA, with an inhibition constant (*K*_I_) of ∼1 nM for statin:enzyme complexes and *K*_M_ of 4 μM [22,23].

Here we describe the development and application of a prenylation block-and-release (PBAR) assay that exploits the ability of mevastatin to reversibly block isoprenoid biosynthesis and allows direct microscopic investigation of the mechanisms of membrane targeting of prenylated GTPases.

## 2 Materials and methods

### 2.1 Reagents

Mevastatin and geranylgeraniol GGOH (Sigma) solutions were prepared in cell culture media. Original stock concentration was 620 μM in ethanol for mevastatin and 500 μM in ethanol for geranylgeraniol. Cycloheximide was used at 2 μg/ml. Antibodies directed against human transferrin receptor (Zymed) and Golgin-97 (Invitrogen) were used at 1:1000 dilution for immunofluorescence.

### 2.2 Cell culture

HeLa cells were maintained in Dulbecco’s Modified Eagle Media (DMEM:F12 (1:1) (Gibco) supplemented with 10% fetal bovine serum (FBS), 2 mM glutamine, 100 U/ml penicillin G and 100 U/ml streptomycin at 37 °C with 10% CO_2_. AtT20 (a murine neuroendocrine pituitary tumor cell line) cells were cultured in DMEM:F12 2:1 with 15% FBS and equal concentrations of glutamine, penicillin G and streptomycin. Immortal Rab27a null melanocyte cell line melan-ash was maintained as described previously [11].

### 2.3 EGFP-GTPase fusion constructs

The construction of vectors pEGFP-Rab23, pEGFP-Rab1a, pEGFP-Rab5, pEGFP-Rab27a and pEGFP-H-Ras were previously described [10–13].

### 2.4 Transfection, immunofluorescence, drug treatments and imaging of cells

Transfection, fixation and immunofluorescence were performed as previously described [11,13]. Drug treatments were initiated 90 min after addition of transfection complexes. This time-point was chosen to begin administration of drugs as the cells are expected to have taken up the plasmid but are unlikely to have targeted significant levels of protein to the endomembrane system. For live cell imaging cells were plated on glass bottomed 30 mm dishes (Matek) and following wash-out of mevastatin were imaged using the 488 nm laser-line of a Zeiss LSM-510 inverted confocal microscope with 100× 1.4NA apochromat oil-immersion objective lens. Images were contrast enhanced using Abode Photoshop CS software and movies were assembled using ImageJ software.

## 3 Results

### 3.1 A mevastatin prenylation block-and-release (PBAR) assay to monitor membrane targeting of prenylated small GTPases in living cells

Our previous studies have investigated the mechanisms by which newly synthesized Rab GTPases are prenylated and targeted to specific intracellular compartments [10–12]. Here we demonstrate the development of a prenylation block-and-release (PBAR) assay that allows the recruitment of prenylated GTPases to specific organelles to be monitored in real-time in living cells using a microscope.

This assay relies upon the ability of mevastatin to act as a reversible inhibitor of HMGCR and thereby block the production of geranylgeranyl (GGPP) and farnesyl (FPP) pyrophosphates, the precursors of the lipid moieties of the prenylation reactions. In turn this blocks the prenylation of newly synthesized substrates of GGT, RGGT and FT (e.g. Rho, Rab and Ras GTPases). Removal of the inhibitor releases this block by allowing GGPP and FPP production and thus prenylation and membrane targeting of unprenylated substrates. The process of membrane targeting from the cytosolic pool of unprenylated protein to the membrane may then be observed directly using fluorescence microscopy.

**Supplementary Fig. 1:**
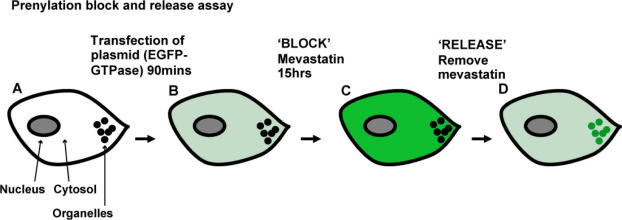
Design of the prenylation block-and-release assay. (A–B) Cells were initially incubated with transfection complexes containing EGFP-GTPase encoding plasmid. Removal of complexes after 90 min and replacement with medium containing mevastatin (B–C ‘BLOCK’) allows uptake of the plasmid and initiation of EGFP-GTPase expression (indicated by pale green color of cytosol in B) but prevents association of EGFP-GTPase with intracellular membranes due to block of prenylation (indicated by black color of organelles in B). Overnight incubation with mevastatin allows cytosolic accumulation of EGFP-GTPase fusion protein (indicated by dark green color of cytosol in C). (C–D ‘RELEASE’) removal of mevastatin containing medium and incubation with normal growth medium allows resumption of prenylation and membrane targeting of the EGFP-GTPase (indicated by transition of cytosol from dark green (C) to pale green (D) and acquisition of green color by cytosolic organelles (D)). In some experiments (Fig. 2 2C) ‘BLOCK’ medium was supplemented by addition of GGPP precursor GGOH to confirm that the mevastatin specifically blocks membrane targeting of EGFP-GTPases by inhibition of prenylation. In other experiments (Fig. 2D) ‘RELEASE’ medium was supplemented with protein synthesis inhibitor cycloheximide to exclude the possibility that organelle associated protein was newly synthesized during the ‘RELEASE’ rather than protein accumulated during the mevastatin ‘BLOCK’.

In more detail, the protocol first involves transfection of cells with plasmid encoding an EGFP-GTPase fusion protein under the control of the strong CMV promoter (Supplementary Fig. 1). This allows expression of a large quantity of fluorescently labeled protein. Shortly post-transfection (90 min) mevastatin is added to cells to prevent prenylation and membrane targeting of newly synthesized EGFP-GTPase thus a pool of unprenylated protein accumulates in the cytosol (Supplementary Fig. 1 ‘BLOCK’). Overnight incubation with mevastatin allows this cytosolic pool of protein to reach a sufficient concentration that it can be easily visualized in transfected cells by confocal fluorescence microscopy and does not appear to affect cell viability. Shorter block times are also possible. Thus on release of mevastatin block and resumption of prenylation, the targeting of cytosolic EGFP-GTPase fusion protein to the membrane of intracellular organelles may be followed in real-time by confocal fluorescence microscopy providing insight into the kinetics and mechanisms by which the newly prenylated proteins are targeted to intracellular membranes (Supplementary Fig. 1 ‘RELEASE’).

### 3.2 Prenylation block-and-release assay applied to study the targeting of newly prenylated EGFP-Rab5a to early endosomes

**Supplementary Fig. 2:**
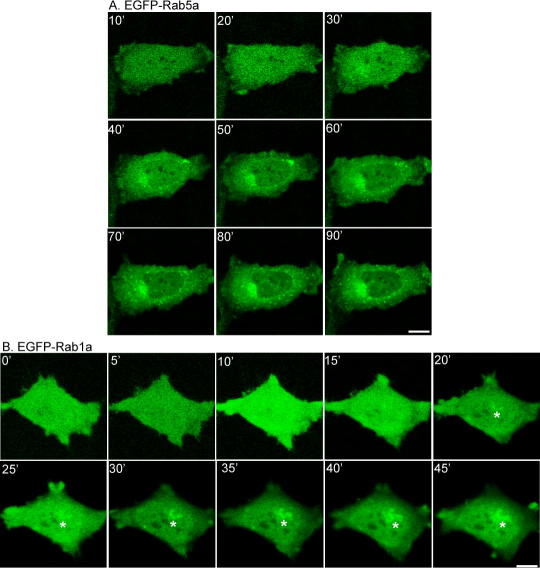
Still images from movies of time-course of Rab membrane targeting following mevastatin prenylation block. HeLa cells expressing EGFP-Rab5a (A) or EGFP-Rab1a (B) in the presence of mevastatin were washed with mevastatin-free medium and images collected at the indicated times (in minutes) following removal of the inhibitor. Images shown in A and B correspond to movies 1 and 2, respectively. Bars = 10 μm.

**Fig. 1:**
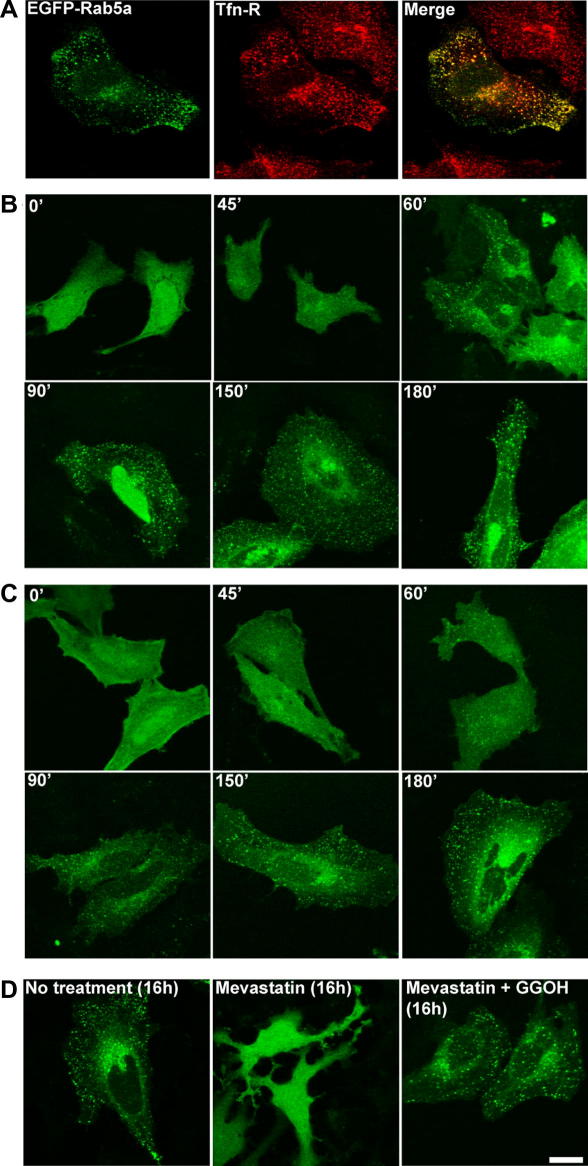
Investigation of the membrane targeting of EGFP-Rab5a using the ‘prenylation block-and-release’ assay. HeLa cells were transfected with pEGFP-Rab5a as described (Section 2) and then treated with drugs as described below to investigate membrane targeting of EGFP-Rab5a. (A) shows EGFP-Rab5a (left) is targeted to Tfn-R (middle) positive early endosomes 24 h after transfection in untreated cells. Co-localization of EGFP-Rab5a (green) and Tfn-R (red) is indicated by yellow color in the right-hand panel. (B) and (C) show images of transfected cells at indicated time-points following mevastatin (10 μM) wash-out in the absence and presence of protein synthesis inhibitor cycloheximide, respectively. (D) confirms the specificity of the mevastatin (20 μM) block on membrane targeting by showing that supplementation of cells with (GGPP precursor) GGOH (10 μM) overcomes the effects of the prenylation inhibitor. Bars = 10 μm. (For interpretation of the references to color in this figure legend, the reader is referred to the web version of this article.)

As a proof of principle we applied this assay to HeLa cells transiently expressing early endosome associated Rab5a [11,24]. As shown in Fig. 1A in the absence of mevastatin treatment we observed efficient targeting of EGFP-Rab5a to transferrin receptor (Tfn-R) early endosomes. In contrast mevastatin treatment of EGFP-Rab5a expressing cells resulted in accumulation of EGFP-Rab5a throughout the nucleus and cytoplasm (Fig. 1B, 0 min). This pattern is similar to the distribution of EGFP alone and differs strikingly from the punctate early endosome associated pattern of EGFP-Rab5a seen in untreated cells (Fig. 1A). This is consistent with the ability of mevastatin to prevent geranylgeranylation and membrane targeting of newly synthesized EGFP-Rab5a. We then released the mevastatin block and used confocal fluorescence microscopy to observe EGFP-Rab5a targeting to early endosomes. As shown in Fig. 1B (60 min) and the Supplementary material (Fig. S2A and Movie 1) in the majority of transfected cells we observed cytosolic EGFP-Rab5a re-distribute within 60 min of removal of inhibitor to a punctate pattern, consistent with targeting to early endosomes. Similar results were achieved when cells were incubated with the protein synthesis inhibitor cycloheximide during the release confirming that the redistribution of EGFP-Rab5a to early endosomes results from restoration of prenylation of the accumulated pool of EGFP-Rab5a rather than targeting of newly synthesized protein (Fig. 1C). Finally to confirm that mevastatin blocks EGFP-Rab5a targeting to early endosome membranes in transfected cells by blocking the production of GGPP we modified the protocol so that post-transfection the membrane permeant GGPP precursor geranylgeraniol (GGOH) was added together with mevastatin. As shown in Fig. 1D targeting of EGFP-Rab5 to early endosomes was normal under these conditions confirming that mevastatin indeed blocks EGFP-Rab5a targeting by specifically blocking GGPP production.

Overall these data indicate that the prenylation block-and-release assay may be used to study the intracellular targeting of prenylated proteins including small GTPases. Regarding Rab5a these data indicate that it is targeted directly from the site of prenylation in the cytosol to the early endosome membrane and does not appear to pass through an intermediate compartment as was reported for targeting of related GTPases of the Ras and Rho families [25,26].

### 3.3 Other newly prenylated Rabs are targeted directly to the organelle on which they reside at steady-state

**Fig. 2:**
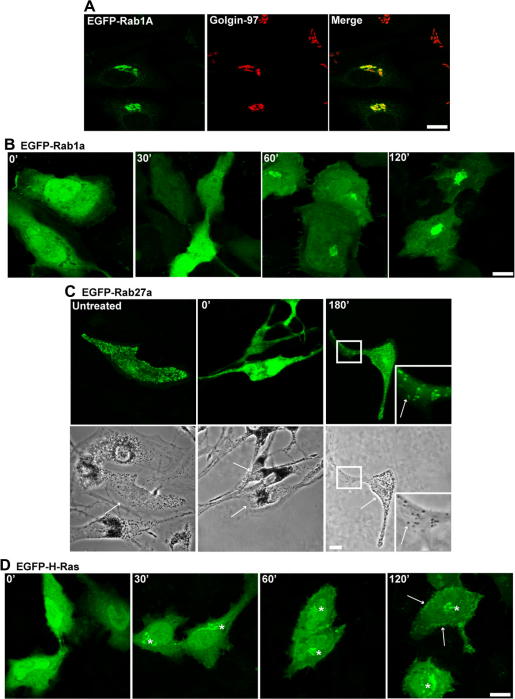
Use of the mevastatin ‘prenylation-block-and-release assay’ to investigate the membrane targeting of EGFP-Rab1a and EGFP-Rab27a. HeLa cells and melan-ash melanocytes were transfected with pEGFP-Rab1a or pEGFP-Rab27a, respectively, treated with mevastatin overnight before the localization of EGFP-fusion protein was monitored during recovery from drug treatment. (A) shows EGFP-Rab1a (left) is targeted to Golgin-97 (middle) positive Golgi membranes in untreated cells. Co-localization of EGFP-Rab1a (green) and Golgin-97 (red) is indicated by yellow in the right-hand panel. (B) shows the localization of EGFP-Rab1a in transfected cells at indicated time-points following mevastatin (10 μM) wash-out. (C) shows the localization of EGFP-Rab27a and melanosomes (upper and lower panels, respectively) in melan-ash melanocytes in the absence, presence or 180 min after wash-out (left, middle and right, respectively) of mevastatin (1 μM). Arrows in phase contrast images indicate transfected cells. For right-hand panels the boxed area is enlarged and shown in the bottom right corner with arrows to indicate co-localization of EGFP-Rab27a with pigmented melanosome. (D) shows the time-course of recovery of membrane targeting of EGFP-H-Ras in HeLa cells following wash-out of mevastatin (10 μM) (white asterisks indicate the position of the Golgi and white arrows indicate plasma membrane associated H-Ras). Bars = 10 μm. (For interpretation of the references to color in this figure legend, the reader is referred to the web version of this article.)

We next used the prenylation block-and-release assay to investigate the targeting of other Rabs to organelles of the secretory pathway. To do this we examined the membrane targeting of EGFP-Rab1a and EGFP-Rab27a that regulate secretory pathway ER to Golgi transport and melanosome docking in peripheral melanocyte dendrites, respectively [13,27]. As for EGFP-Rab5a, we observed that in the untreated cells EGFP-Rab1a and EGFP-Rab27a were correctly targeted to the Golgi stack and mature pigmented melanosomes in melanocytes, respectively (Fig. 2A and C). Moreover, we confirm the functionality of EGFP-Rab27a as its association with melanosomes restores their peripheral retention in Rab27a null (melan-ash) melanocytes (Fig. 2C) [11]. Mevastatin treatment of transfected cells resulted in a diffuse distribution of both EGFP-Rab1a and EGFP-Rab27a throughout the nucleus and cytoplasm consistent with the disruption of prenylation and membrane targeting (Fig. 2B and C). Also in the case of EGFP-Rab27a in melan-ash function is disrupted as evidence by maintenance of perinuclear clustered melanosomes in transfected cells (Fig. 2C). Subsequent removal of mevastatin resulted in membrane targeting of the newly prenylated protein. For EGFP-Rab1a protein was targeted from the cytosol to a ribbon-like perinuclear structure, consistent with the normal distribution of the Golgi stack, (Fig. 2B and Supplementary Fig. 2B and Movie 2) while EGFP-Rab27a was targeted from the cytosol to pigmented melanosomes and restored their retention in peripheral dendrites consistent with full functionality of the targeted protein (Fig. 2C). Interestingly while both Rabs targeted directly to these organelles we observed differences in the kinetics of recovery from the block for each Rab with EGFP-Rab1a and EGFP-Rab27a requiring 30 and 180 min, respectively. In contrast, following release of the mevastatin block EGFP-H-Ras was first observed on the Golgi complex within 30 min then accumulated at the plasma membrane at later time-points (Fig. 2D). This is consistent with previous reports showing that H-Ras is targeted to the plasma membrane via the classical secretory pathway [25].

### 3.4 Prenylation pathway influences the sensitivity to mevastatin block and the targeting kinetics of prenylated GTPases

**Fig. 3:**
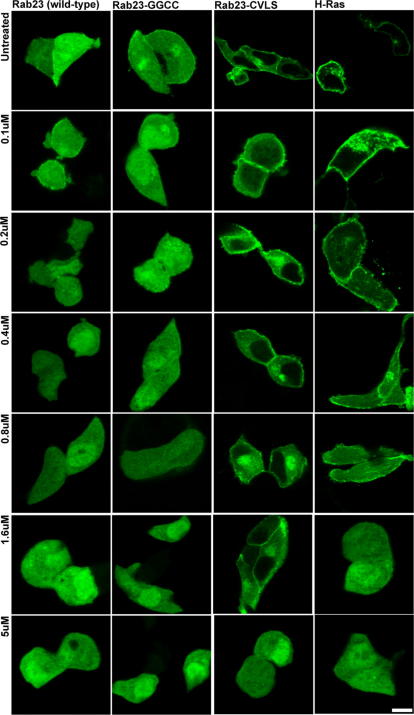
Investigation of the sensitivity of different prenylation pathways to disruption by mevastatin. AtT20 cells were transiently transfected with plasmids allowing the expression of the indicated EGFP-fusion protein, incubated overnight with the indicated concentration of mevastatin and fixed before the intracellular localization of the heterologously expressed protein was observed using confocal fluorescence microscopy. Bars = 10 μm.

**Fig. 4:**
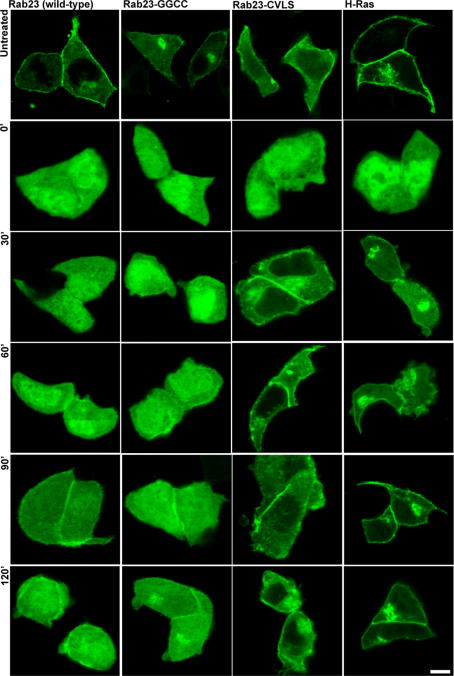
Investigation of the membrane targeting kinetics of GTPases that are substrates of different prenylation pathways following mevastatin wash-out. AtT20 cells were transiently transfected with plasmids allowing the expression of the indicated EGFP-fusion protein and incubated overnight with 5 μM mevastatin. The intracellular localization of the heterologously expressed protein was observed using confocal fluorescence microscopy at the indicated time-points following mevastatin wash-out. Bars = 10 μm.

Finally we investigated whether the type (farnesyl versus geranylgeranyl) i.e. prenylation by different prenyl transferases, or the number of lipids (one or two) associated with a GTPase influences sensitivity to mevastatin and the mechanism of membrane targeting. As mentioned above most Rabs contain two prenylatable cysteine residues at their extreme C-terminus and are di-geranylgeranylated by RGGT [4]. However, a significant number including Rab23 have only one such cysteine. Also as described in the introduction other small GTPases e.g. Rho and Ras proteins, are singly prenylated by either GGT or FT. To address these issues we transfected mouse pituitary derived cell line (AtT20) with EGFP-H-Ras, EGFP-Rab23 (C-terminus tetra-peptide CSVP), predicted to be singly prenylated by FT and RGGT, respectively, and two Rab23 mutants; EGFP-Rab23-GGCC, that may be doubly prenylated by RGGT and EGFP-Rab23-CVLS, predicted to be a substrate for FT. We then tested the mevastatin sensitivity of prenylation and cytosol to membrane targeting of each of these proteins. As previously reported we found that all four proteins were targeted to the plasma membrane in the absence of mevastatin [25,28] (Fig. 3 untreated). Meanwhile addition of different concentrations of mevastatin revealed that targeting of Rab23 and Rab23-GGCC mutant (0.1 μM mevastatin disrupts targeting) were more sensitive to mevastatin than either Rab23-CVLS or H-Ras (5 μM and 1.6 μM mevastatin disrupt targeting, respectively). These data indicate that prenylation/targeting of FT substrates is less sensitive to mevastatin than RGGT substrates. We next investigated the recovery kinetics of prenylation/membrane targeting of GTPases that are substrates of different prenyltransferases. Consistent with the higher mevastatin sensitivity of RGGT compared with FT we found that recovery of cytosol to plasma membrane targeting was significantly slower for RGGT substrates, Rab23 wild-type and Rab23-GGCC, than for FT substrates Rab23-CVLS and H-Ras (Fig. 4, 90 min versus 30 min, respectively).

## 4 Discussion

Here we established a novel prenylation block-and-release assay that allows direct morphological investigation of the intracellular targeting of prenylated GTPases in living cells. We exploited the ability of mevastatin to reversibly inhibit HMGCR and thus reversibly block production of GGPP and FPP so preventing the prenylation and membrane targeting of prenylated proteins. Release of the block, on removal of the drug, allows resumption of prenylation and membrane targeting. Coupling this treatment with over-expression of fluorescently labeled protein provides a large pool of unprenylated protein whose cytosol to membrane targeting may then be observed on removal of the drug. Using this assay we report several new findings regarding membrane targeting of Ras family small GTPases.

Firstly, we observed that different Rabs have differing kinetics of cytosol to membrane targeting after release of the mevastatin block. For instance in HeLa cells Rab1a required less than 30 min to target to the Golgi but Rab5a required 60 min to target early endosomes while in melanocytes Rab27a required 180 min to target melanosomes. One possibly explanation for the differing rate of targeting between different Rabs in same cell type might be that the prenylation machinery more efficiently modify certain Rabs. In support of this possibility several studies suggest that Rab27a is less efficiently prenylated than other Rabs including Rab1a [29]. Interestingly we also observed that H-Ras cytosol to plasma membrane targeting during the release is more rapid in AtT20 than HeLa (30 versus 120 min, respectively). Together with the differences in kinetics of targeting of Rabs in different cell types (e.g. Rab27a in melanocytes versus Rab1a in HeLa cells) these observations suggest that rates of GGPP and FPP production as well as expression levels of prenyltransferases may vary between these cell types.

Secondly, of the Rabs tested we observed that all appeared to be targeted from the cytosol directly to the membrane/organelle on which they reside at steady-state and did not traffic there via an intermediate location. This is consistent with a previous report in which Rab5 and Rab7 in complex with REP1 were added to streptolysin-O permeabilized cells and shown to target directly to either early or late endosomes [16]. But is in contrast to previous reports on the targeting of Ras and Rho family GTPases which reveal that several of these proteins are initially targeted to ER where they are modified by Rce and Icmt before passing through classical and non-classical secretory pathways to the plasma membrane [25,26]. Similarly we previously reported that Rab23 and other CAAX containing Rab proteins are post-prenylation modified *in vivo* by Rce1 and Icmt, resident at the ER [10]. Although the physiological significance of post-prenylation processing is unresolved this modification may increase stability of membrane association of Rab23. This observation led us to propose that CAAX containing Rabs, such as Rab23, may be initially targeted post-prenylation to the ER for processing followed by transfer to the plasma membrane. However, in this study we found that Rab23 appears to target directly to the plasma membrane without prior accumulation on endo-membranes such as ER. One caveat to this is that the cytosolic accumulation of unprenylated EGFP-Rab23 may obscure transient localization of the protein to the ER membrane.

Thirdly, we found that GTPases that are substrates of different prenyl transferases are differentially sensitive to mevastatin. In particular we found RGGT substrates are more sensitive to mevastatin and take longer to recover from mevastatin treatment than are FT substrates. One possible explanation for this observation is that there might be a larger reserve of FPP and FT than GGPP GGT in the cell. Also farnesyl is a precursor of geranylgeranyl therefore its synthesis is more likely to resume rapidly after the withdrawal of mevastatin.

Fourthly, we observed using Rab23 as an example that the number of prenylatable cysteine present in the C-terminus of a GTPase does not strongly affect its sensitivity to/or the kinetics of its recovery from mevastatin treatment. This is consistent with the likelihood that all Rabs are prenylated via RGGT regardless of whether they contain one or two prenylatable cysteine residues and therefore rate of prenylation and membrane targeting is dependent upon the activity of the RGGT pathway.

## 5 Conclusion

We utilized the ability of Statins to block the prenylation of proteins to visualize membrane targeting of small GTPases. The assay revealed that prenylated proteins have variable susceptibility to mevastatin inhibition and variable time of recovery indicating a hierarchy of the picking order of the prenylated proteins. We propose that this assay may be used for further studies (e.g. targeting, biological functions and mechanisms) of the membrane targeting of the many the prenylated proteins.

## Appendix A Supplementary data

Movie 1 Recovery of membrane targeting of EGFP-Rab5a in mevastatin treated HeLa cells following the removal of inhibitor. Images (total of 40) were collected every 120 s minutes playback speed is 10 s^−1^.

Movie 2 Recovery of membrane targeting of EGFP-Rab1a in mevastatin treated HeLa cells following the removal of inhibitor. Images (total of 90) were collected every 30 s minutes playback speed is 10 s^−1^.

## References

[1] Casey P.J., Seabra M.C. (1996). Protein prenyltransferases. J. Biol. Chem..

[2] Hancock J.F., Magee A.I., Childs J.E., Marshall C.J. (1989). All ras proteins are polyisoprenylated but only some are palmitoylated. Cell.

[3] Seabra M.C. (1998). Membrane association and targeting of prenylated Ras-like GTPases. Cell Signal..

[4] Leung K.F., Baron R., Seabra M.C. (2006). Thematic review series: lipid posttranslational modifications. Geranylgeranylation of Rab GTPases. J. Lipid Res..

[5] Perez-Sala D. (2007). Protein isoprenylation in biology and disease: general overview and perspectives from studies with genetically engineered animals. Front. Biosci..

[6] Wright L.P., Philips M.R. (2006). Thematic review series: lipid posttranslational modifications. CAAX modification and membrane targeting of Ras. J. Lipid Res..

[7] Pechlivanis M., Kuhlmann J. (2006). Hydrophobic modifications of Ras proteins by isoprenoid groups and fatty acids – more than just membrane anchoring. Biochim. Biophys. Acta.

[8] Pereira-Leal J.B., Hume A.N., Seabra M.C. (2001). Prenylation of Rab GTPases: molecular mechanisms and involvement in genetic disease. FEBS Lett..

[9] Stenmark H. (2009). Rab GTPases as coordinators of vesicle traffic. Nat. Rev. Mol. Cell. Biol..

[10] Leung K.F., Baron R., Ali B.R., Magee A.I., Seabra M.C. (2007). Rab GTPases containing a CAAX motif are processed post-geranylgeranylation by proteolysis and methylation. J. Biol. Chem..

[11] Ali B.R., Wasmeier C., Lamoreux L., Strom M., Seabra M.C. (2004). Multiple regions contribute to membrane targeting of Rab GTPases. J. Cell Sci..

[12] Gomes A.Q., Ali B.R., Ramalho J.S., Godfrey R.F., Barral D.C., Hume A.N., Seabra M.C. (2003). Membrane targeting of Rab GTPases is influenced by the prenylation motif. Mol. Biol. Cell.

[13] Hume A.N., Collinson L.M., Rapak A., Gomes A.Q., Hopkins C.R., Seabra M.C. (2001). Rab27a regulates the peripheral distribution of melanosomes in melanocytes. J. Cell Biol..

[14] Wilson A.L., Erdman R.A., Castellano F., Maltese W.A. (1998). Prenylation of Rab8 GTPase by type I and type II geranylgeranyl transferases. Biochem. J..

[15] Pereira-Leal J.B., Seabra M.C. (2000). The mammalian Rab family of small GTPases: definition of family and subfamily sequence motifs suggests a mechanism for functional specificity in the Ras superfamily. J. Mol. Biol..

[16] Alexandrov K., Horiuchi H., Steele-Mortimer O., Seabra M.C., Zerial M. (1994). Rab escort protein-1 is a multifunctional protein that accompanies newly prenylated rab proteins to their target membranes. EMBO J..

[17] Istvan E.S., Deisenhofer J. (2001). Structural mechanism for statin inhibition of HMG-CoA reductase. Science.

[18] Endo A. (1992). The discovery and development of HMG-CoA reductase inhibitors. J. Lipid Res..

[19] Roth B.D. (2002). The discovery and development of atorvastatin, a potent novel hypolipidemic agent. Prog. Med. Chem..

[20] Poynter J.N., Gruber S.B., Higgins P.D., Almog R., Bonner J.D., Rennert H.S., Low M., Greenson J.K., Rennert G. (2005). APC I1307K and the risk of prostate cancer. N. Engl. J. Med..

[21] Walker K., Olson M.F. (2005). Targeting Ras and Rho GTPases as opportunities for cancer therapeutics. Curr. Opin. Genet. Dev..

[22] Corsini A., Maggi F.M., Catapano A.L. (1995). Pharmacology of competitive inhibitors of HMG-CoA reductase. Pharmacol. Res..

[23] Bischoff K.M., Rodwell V.W. (1992). Biosynthesis and characterization of (S)-and (R)-3-hydroxy-3-methylglutaryl coenzyme A. Biochem. Med. Metab. Biol..

[24] Gorvel J.P., Chavrier P., Zerial M., Gruenberg J. (1991). Rab5 controls early endosome fusion in vitro. Cell.

[25] Choy E., Chiu V.K., Silletti J., Feoktistov M., Morimoto T., Michaelson D., Ivanov I.E., Philips M.R. (1999). Endomembrane trafficking of ras: the CAAX motif targets proteins to the ER and Golgi. Cell.

[26] Michaelson D., Silletti J., Murphy G., D’Eustachio P., Rush M., Philips M.R. (2001). Differential localization of Rho GTPases in live cells: regulation by hypervariable regions and RhoGDI binding. J. Cell Biol..

[27] Tisdale E.J., Bourne J.R., Khosravi-Far R., Der C.J., Balch W.E. (1992). GTP-binding mutants of rab1 and rab2 are potent inhibitors of vesicular transport from the endoplasmic reticulum to the Golgi complex. J. Cell Biol..

[28] Evans T.M., Ferguson C., Wainwright B.J., Parton R.G., Wicking C. (2003). Rab23, a negative regulator of hedgehog signaling, localizes to the plasma membrane and the endocytic pathway. Traffic.

[29] Larijani B., Hume A.N., Tarafder A.K., Seabra M.C. (2003). Multiple factors contribute to inefficient prenylation of Rab27a in Rab prenylation diseases. J. Biol. Chem..

