# Supplementary figures and images for: A novel statin-mediated “prenylation block-and-release” assay provides insight into the membrane targeting mechanisms of small GTPases
Source: Biochem Biophys Res Commun. 2010 Jun 18;397(1):34–41. doi: 10.1016/j.bbrc.2010.05.045 (PMC2908739; doi:10.1016/j.bbrc.2010.05.045)

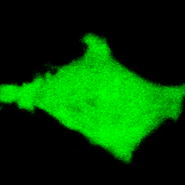

Supplement: Movie 2 — Recovery of membrane targeting of EGFP-Rab1a in mevastatin treated HeLa cells following the removal of inhibitor. Images (total of 90) were collected every 30 s minutes playback speed is 10 s−1. [file mmc2.jpg]
